# Advanced Access scheduling in general practice and use of primary care: a Danish population-based matched cohort study

**DOI:** 10.3399/bjgpopen20X101091

**Published:** 2020-11-04

**Authors:** Maria Bang, Henrik Schou Pedersen, Bodil Hammer Bech, Claus Høstrup Vestergaard, Jannik Falhof, Hans Christian Kjeldsen, Peter Vedsted, Mogens Vestergaard

**Affiliations:** 1 Reseach Unit for General Practice, Aarhus, Denmark; 2 Department of Public Health, Aarhus University, Aarhus, Denmark

**Keywords:** primary health care, general practice, after-hours care, health services accessibility, Denmark

## Abstract

**Background:**

Advanced access scheduling (AAS) allows patients to receive care from their GP at the time chosen by the patient. AAS has shown to increase the accessibility to general practice, but little is known about how AAS implementation affects the use of in-hours and out-of-hours (OOH) services.

**Aim:**

To describe the impact of AAS on the use of in-hours and OOH services in primary care.

**Design & setting:**

A population-based matched cohort study using Danish register data.

**Method:**

A total of 161 901 patients listed in 33 general practices with AAS were matched with 287 837 reference patients listed in 66 reference practices without AAS. Outcomes of interest were use of daytime face-to-face consultations, and use of OOH face-to-face and phone consultations in a 2-year period preceding and following AAS implementation.

**Results:**

No significant differences were seen between AAS practices and reference practices. During the year following AAS implementation, the number of daytime face-to-face consultations was 3% (adjusted incidence rate ratio [aIRR] = 1.03; 95% confidence interval [CI] = 0.99 to 1.07) higher in the AAS practices compared with the number in the reference practices. Patients listed with an AAS practice had 2% (aIRR = 0.98; 95% CI = 0.92 to 1.04) fewer OOH phone consultations and 6% (aIRR = 0.94; 95% CI = 0.86 to 1.02) fewer OOH face-to-face consultations compared with patients listed with a reference practice.

**Conclusion:**

This study showed no significant differences following AAS implementation. However, a trend was seen towards slightly higher use of daytime primary care and lower use of OOH primary care.

## How this fits in

AAS is intended to improve the access to primary care. This population-based cohort study is the first to explore how AAS affects the use of in-hours and OOH primary care. The presented results indicate no significant changes in the healthcare utilisation following AAS implementation. However, a trend was seen towards slightly higher use of daytime primary care and lower use of OOH primary care. The influence of AAS on the use of primary care should be further explored in future studies.

## Introduction

Accessibility and continuity of care are two cardinal goals of primary care. Many GPs struggle to achieve these goals owing to an increasing number of patient visits.^1–3^ Reduced access to daytime primary care can lead to worse health outcomes as well as physical and emotional stress in the patient.^4^ Moreover, the patients may choose to contact the OOH services or emergency departments (ED).^5–8^

To increase accessibility and ensure continuity of care, many GPs have introduced AAS.^9^ AAS is based on the principle that the patient can choose the date to be seen by the GP.^10^ Traditional scheduling systems are based on advance booking of appointments, where only a little time is reserved for urgent care.^11^ AAS requires no distinction between urgent and routine care, and patient preferences determine when appointments take place.^12^ Six key elements of AAS are important for successful application: balancing supply and demand; reducing backlog; reducing the variety of appointment types; developing contingency plans for unusual circumstances; adjusting demand profiles; and increasing ‘bottleneck’ resources.^1^ AAS has been shown to increase the accessibility of primary care,^4,13,14^ to reduce no-shows, to increase patient and provider satisfaction, and to ensure continuity of care.^15–18^

The effect of AAS on healthcare utilisation is still inconclusive, especially the effect on the use of OOH services. Solberg *et al* found that the use of daytime primary care increased following implementation of AAS, while the use of urgent care decreased significantly.^19^ Hudec *et al* found a significant drop in ED visits following AAS implementation,^16^ while Subramanian *et al* found no such correlation.^20^

The present study aimed to describe the impact of AAS on the use of daytime and OOH primary care in the year preceding and following AAS implementation in a number of Danish general practices, compared with a reference group of practices without AAS. It was hypothesised that the increased accessibility through AAS during office hours would cause higher use of daytime primary care and lower use of OOH primary care.

## Method

### Study design

A population-based matched cohort study was conducted, including patients listed with a general practice with AAS and their matched patients listed with a practice without AAS. Background information about the study population and their healthcare utilisation was extracted from Danish registers. This information was linked at a personal level through the unique personal identification number (PIN), which is assigned to all Danish citizens at birth or immigration.^21^

### Study population

An internet search identified general practices using AAS according to their website (*n* = 48). Practices in the Capital Region of Denmark (*n* = 10) were not included owing to missing OOH data for this region in the Danish National Health Service Register (NHSR),^22^ leaving 38 practices to be invited. Thirty-three practices with AAS consented to participate in the study. Each included AAS practice was matched with two reference practices without AAS. The reference practices were identified in the Patient List Database^23^ based on municipality and number of listed patients. The date of AAS implementation was defined as the index date for the AAS practice and the two matched reference practices.

Most of the Danish population (98%) is listed with a specific general practice, which must be consulted for medical advice.^21^ All patients listed with a participating AAS practice or reference practice at the index date were included in the study. A total of 449 738 patients were included, of whom 161 901 were listed with an AAS practice. The cohort was followed until death, emigration, change of GP, or the end of study (that is, 1 year following the index date or 30 June 2018), whichever came first.

### Exposure

All participating AAS practices were initially interviewed. A brief questionnaire was used to verify their use of AAS and confirm the index date. The data were collected from 1 January 2018–31 March 2018 by phone or email, depending on the preference of the practice. Anonymous data extracted from the Patient List Database were used to identify the reference practices, and the staff in these practices were not interviewed.

### Outcome

Contacts to in-hours and OOH primary care in the year preceding and following the index date were applied as outcome measures. This included daytime face-to-face consultations, OOH face-to-face consultations, and OOH phone consultations. Information on all services provided in general practice was obtained from the NHSR^22^ (see Supplementary Appendix S1).

### Covariates

The Danish Civil Registration System (CRS)^21^ contained updated information about the patient’s sex, age, and vital status. Statistics Denmark^24^ provided information about migration, ethnic group (Danish, immigrants, and descendants of immigrants), cohabitation status (cohabiting, living alone, children living at home, and children not living at home), family income, and highest attained education level (≤10 years, 11–15 years, and ≥16 years). Information about calendar time was obtained from the index date identified from the questionnaires. The Charlson Comorbidity Index (CCI) was used to adjust for a range of comorbid conditions.^25^ Information about these conditions was obtained from the Danish National Patient Registry (DNPR)^26^ and the Danish Cancer Registry.^27^

### Statistical analysis

All data were analysed using both unadjusted and adjusted negative binomial regression models with cluster-robust variance estimation at practice level. These models yielded incidence rate ratios (IRRs) and 95% CIs with negative binomial distribution. The outcomes were analysed in pre-specified time periods around the index date. In the main analyses, consultations conducted in the entire year preceding and following the index date were included. The year following the implementation of AAS was subsequently divided into 3-month periods to explore if the use of healthcare services varied throughout the year. In the adjusted models, sex, age, calendar time, month of year, ethnic group, cohabitation status, family income, highest attained education level, and CCI were adjusted for. All covariates were measured at the index date.

## Results

A total of thirty-three general practices with AAS were included in this study and matched with 66 reference practices. The study included 449 738 patients, of whom 161 901 (36%) were listed with an AAS practice (Table 1). The differences between the two groups were small, although the patients listed with an AAS practice were more often men, less educated, had lower income, and were more often immigrants or descendants of immigrants compared with patients from the reference practices (Table 1).

**Table 1.** Characteristics of study population at index date

	**Patients in AAS practices, *n* (%)**	**Patients in references practices**, *n* (%)****
***N* (%**)	161 901 (36.0)	287 837 (64.0)
**Clinic size, mean (SD**)	4906 (1902.2)	4361 (1606.0)
**Mean age, years (SD**)	41.52 (23.49)	41.76 (23.54)
**Sex**		
Male	82 671 (51.1)	145 101 (50.4)
Female	79 230 (48.9)	142 736 (49.6)
**Education level**		
≤10 years	46 178 (28.5)	80 727 (28.1)
>10 and ≤15 years	61 117 (37.8)	109 013 (37.9)
>15 years	25 825 (16.0)	46 523 (16.2)
Unknown	28 781 (17.7)	51 575 (17.8)
**Cohabitation status**		
Cohabiting	80 398 (49.7)	142 777 (49.6)
Living alone	34 067 (21.0)	61 032 (21.2)
Children living at home	32 078 (19.8)	57 327 (19.9)
Children not living at home	15 358 (9.5)	26 701 (9.3)
**Family income**		
Low	55 095 (34.0)	93 870 (32.6)
Medium	54 032 (33.4)	95 464 (33.2)
High	52 774 (32.6)	98 503 (34.2)
**Ethnic group**		
Danish	146 473 (90.5)	262 050 (91.0)
Immigrant	12 146 (7.5)	20 485 (7.1)
Descendants of immigrants	3282 (2.0)	5302 (1.9)
**Calendar time**		
January 2011–December 2015	54 282 (33.5)	98 532 (34.2)
January 2016–December 2016	61 822 (38.2)	106 132 (36.9)
January 2017–December 2017	45 797 (28.3)	83 173 (28.9)
**Number of chronic conditions**		
0	127 580 (78.8)	226 628 (78.7)
1–2	27 787 (17.2)	49 416 (17.2)
≥3	6534 (4.0)	11 793 (4.1)

AAS advanced access scheduling SD standard deviation

**Figure 1.**
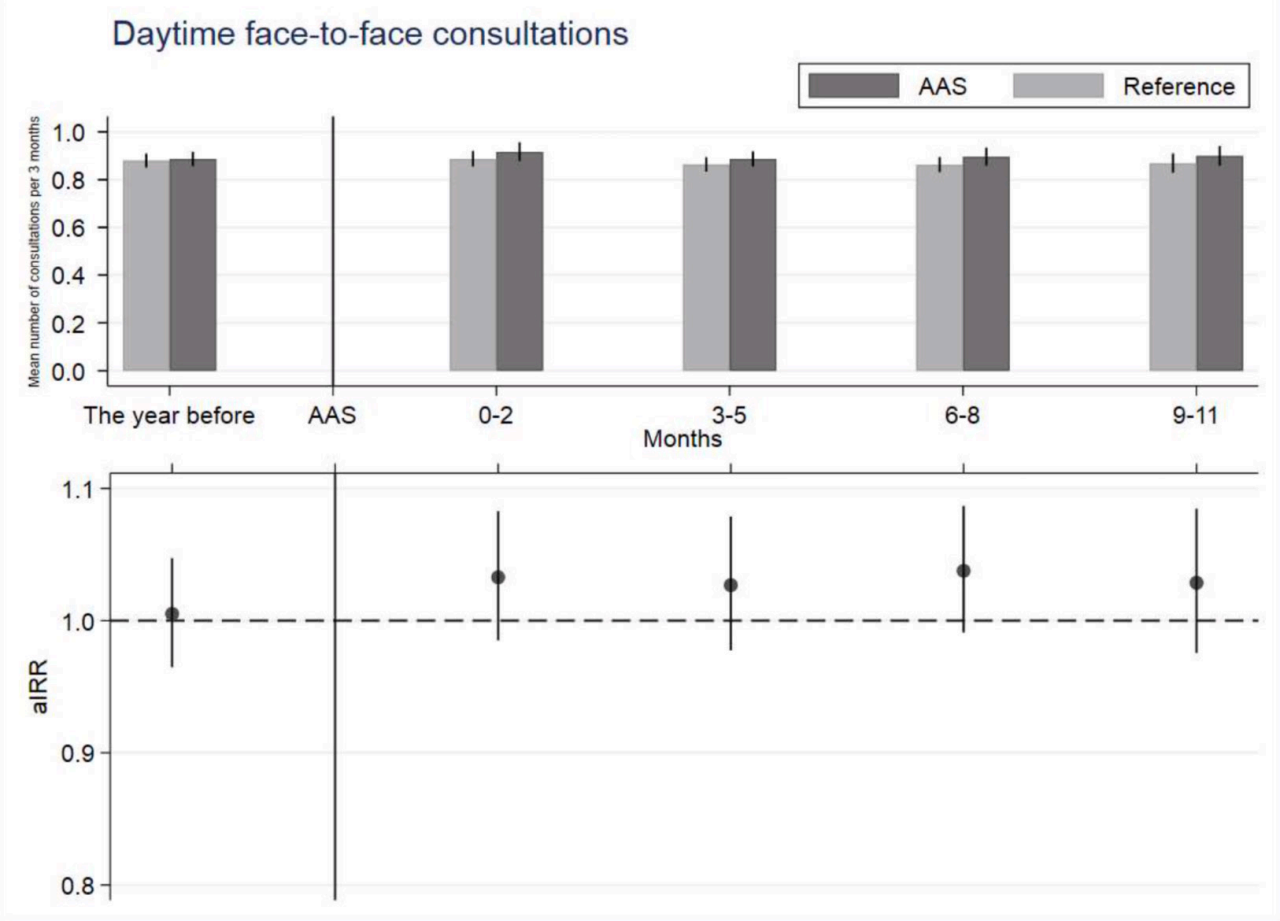
Daytime face-to-face consultations in general practice 1 year before and 1 year after the index date. The vertical line marks the index date. Upper part: mean number of consultations per patient per 3 months with 95% confidence intervals (CIs) for patients listed with advanced access scheduling (AAS) practices and reference practices. Lower part: adjusted incident rate ratios (aIRRs) with 95% CIs adjusted for sex, age, vital status, ethnic group, cohabitation status, family income, highest-achieved education level, Charlson Comorbidity Index, calendar time, and month; all measured at the index date.

Following AAS implementation, the patients listed in an AAS practice had slightly more face-to-face consultations compared with the references, although the difference did not reach statistical significance (figure 1, upper part). The adjusted incidence rate ratio (aIRR) was 1.03 (95% CI = 0.99 to 1.07) in the year following AAS implementation. No difference was seen in the aIRR when the year was divided into 3-month intervals Figure 1, lower part). In the year preceding ASS implementation, the aIRR was 1.01 (95% CI = 0.96 to 1.05) when the patients listed in an AAS practice were compared with the references.

**Figure 2.**
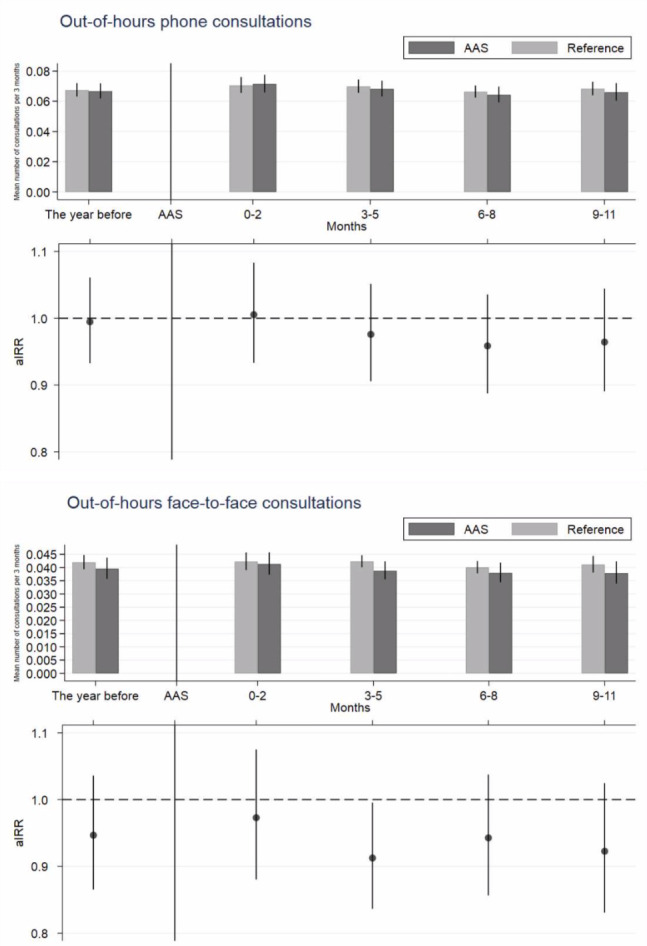
Out-of-hours face-to-face and phone consultations 1 year before and 1 year after the index date. The vertical line marks the index date. Upper part: mean number of consultations per patient per 3 months with 95% confidence intervals (CIs) for patients listed with advanced access scheduling (AAS) practices and reference practices. Lower part: adjusted incident rate ratios (aIRRs) with 95% CIs adjusted for sex, age, vital status, ethnic group, cohabitation status, family income, highest-achieved education level, Charlson Comorbidity Index, calendar time, and month; all measured at the index date.

The patients listed in an AAS practice had fewer OOH consultations than the references (Figure 2, lower part), but these results were not statistically significant. In the year following AAS implementation, the aIRR was 0.98 (95% CI = 0.918 to 1.041) for phone consultations and 0.94 (95% CI = 0.863 to 1.021) for face-to-face consultations when the AAS patients were compared with the references. When dividing the year into 3-month intervals, no difference between the periods was found (Figure 2, lower part). In the year preceding AAS implementation, the aIRR was 1.00 (95% CI = 0.93 to 1.06) for phone consultations and 0.95 (95% CI = 0.87 to 1.04) for face-to-face consultations when the AAS patients were compared with the references (Figure 2, upper part).

## Discussion

### Summary

In this large population-based cohort study, it was found that the patients listed with an AAS practice consulted their GP slightly more during the daytime and requested slightly fewer OOH services following the implementation of AAS compared with the references. However, the findings were not statistically significant.

### Strengths and limitations

To the authors' knowledge, this is the largest study to evaluate AAS and the association with use of daytime and OOH primary care. Previous studies comprised only up to six AAS practices,^14,16,20^ were based on selected study populations,^19,20^ or included no reference practices.^19^ A nationwide cohort of all patients listed with a general practice offering AAS has been established and they have been matched with patients in reference practices without AAS. The matching of practices was based on the patient population and the geographical location of the practices. The cohort was followed for up to 1 year following AAS implementation and had complete follow-up.

The AAS practices were identified by a systematic and thorough internet search, but it cannot be excluded that some practices were missed. However, an incomplete registration of AAS practices would have affected the results only if their patients had different patterns of healthcare utilisation, which there is no reason to believe would be the case. All identified AAS practices were asked to verify (in a short questionnaire) their use of AAS, including the index date. Some of the practices had implemented the AAS system several years earlier. Thus, the information given in the questionnaires, such as the index date, may have been affected by recall bias. Imprecise information about the index date would have affected the results as this date was used to estimate the effect of AAS implantation in the analyses.

The results showed that the use of OOH face-to-face consultations was lower for the patients listed in an AAS practice in the year preceding AAS implementation. This suggests that these practices might have differed from the reference practices at other parameters already preceding AAS implementation, or that the index date is imprecise because AAS was implemented over a period of time rather than on an exact date. Anonymous data extracted from the Patient List Database was used to identify the reference practices. The reference practice staff was not interviewed, and it cannot be excluded that some of the reference practices might have used a scheduling system ensuring equally high accessibility as the AAS system, which could have caused the results to underestimate the true effect of AAS.

In the analyses, adjustments were made for relevant covariates based on information from the CRS,^21^ Statistics Denmark,^24^ the DNPR,^26^ and the Danish Cancer Registry,^27^ and the index date was based on questionnaire responses. This approach was taken to reduce the risk of confounding from unmeasured factors, but the risk of residual confounding cannot be excluded. The study did not adjust for information about type of practice, and the number of working doctors and nurses was not included in the adjustment since the authors did not have this information. This could have affected the result if major differences existed between the AAS practices and the reference practices. The data on healthcare utilisation was obtained from electronic records in the NHSR, and this data did not rely on the memory of the GPs or the patients. The NHSR registrations of contacts to the GPs^22^ are considered highly valid as the GPs are reimbursed on the basis of these registrations following validation by the Danish regions. The authors see no reason why the quality of the healthcare utilisation registrations should be different between AAS practices and reference practices. Therefore, misclassification is likely to be non-differential, which tends to bias the study results towards the null hypothesis.

### Comparison with existing literature

Some studies have shown that the implementation of AAS increases the use of daytime primary care^19,27^ and healthcare expenses.^16^ In keeping with previous studies, the results showed higher numbers of daytime consultations following AAS implementation.^28^ It was expected that the number of daytime face-to-face consultations would increase even more, especially in the period immediately following the implementation. However, this was not the case. In line with expectations, the study found a small reduction in the use of OOH services. Converted to absolute numbers, the patients listed with an AAS practice had 2716 fewer OOH face-to-face consultations and 1657 fewer OOH phone consultations compared with the references in the year following AAS implementation.

This study was the first to evaluate the use of OOH services following AAS implementation. Other studies have found a reduction in the use of EDs^16^ and urgent care clinics.^19^ However, these studies are not directly comparable to the present study as they had different outcomes and were conducted in countries with a different healthcare system to the Danish system. The results of the present study can be generalised to other countries with a healthcare system comparable to the Danish.^29^ However, the results might have been different if the study had been conducted in a country without free and equal access to health care.

A meta-analysis from 2017 found that AAS implementation reduced the waiting time for a primary care appointment by 11.3 days.^4^ Limited access to daytime primary care has previously been associated with higher use of EDs and OOH services.^5–7,29,30^ Moreover, studies have shown that patients experiencing difficulty with getting an appointment with their GP tend to use EDs and OOH services instead.^6,7,30^ Zhoe *et al* estimated that the use of OOH services can be reduced by 11% if the access to daytime primary care is optimal.^5^ Other studies have triaged the contacts to EDs and OOH services according to urgency, and found that it should be possible to reduce the number of non-urgent contacts by ensuring better access to daytime primary care.^6,31^ Unfortunately, the data did not include sufficient information to make a division between urgent and non-urgent contacts to the OOH services.

AAS implementation has been shown to be difficult in some practices. A large study set in England found that only 161 AAS practices ─ and less than half of the practices claiming to operate AAS ─ used all the key principles of the AAS model.^1,32^ Other studies have shown that AAS implementation was very difficult and that the desired benefits from the AAS system were not obtained.^33–35^ Factors such as patient–provider satisfaction^16–18,27^ and continuity of care^36–38^ have shown to vary following AAS implementation. A large-scale study conducted in 47 general practices in the UK found that a 10% increase in the proportion of AAS appointments was associated with an 8% decrease in patient satisfaction.^39^

AAS appointments have been found to be preferred by younger patients, whereas older patients seem to favour pre-booked appointments with their usual GP, especially in case of chronic disease checks.^13,37,39^ The right balance between demand and supply of appointments seems to be key when meeting the patient’s needs for access to care and continuity in care.^35,40^ In the present study, the access to primary care was investigated as an organisational concept, whereas other aspects of access to health care were not investigated. Access to a healthcare system is a multidimensional construct, and patients’ individual characteristics are known to affect their healthcare-seeking behaviour.^41,42^

### Implications for research and practice

This study is the first to investigate the influence of AAS implementation on the use of primary care in Denmark. The findings present new knowledge to clinicians and may be generalisable to comparable countries with universal access to health care. Further research is needed to explore whether AAS implementation may influence the use of specific types of contacts to the OOH services, such as non-urgent contacts.
